# Porous Hydrogels for Immunomodulatory Applications

**DOI:** 10.3390/ijms25105152

**Published:** 2024-05-09

**Authors:** Cuifang Wu, Honghong Zhang, Yangyang Guo, Xiaomin Sun, Zuquan Hu, Lijing Teng, Zhu Zeng

**Affiliations:** 1 Key Laboratory of Infectious Immune and Antibody Engineering in University of Guizhou Province, Engineering Research Center of Cellular Immunotherapy of Guizhou Province, School of Basic Medical Sciences/School of Biology and Engineering (School of Modern Industry for Health and Medicine), Guizhou Medical University, Guiyang 550025, China; cuifang_wu@163.com (C.W.);; 2 Immune Cells and Antibody Engineering Research Center in University of Guizhou Province, Key Laboratory of Biology and Medical Engineering, Guizhou Medical University, Guiyang 550025, China; 3 State Key Laboratory of Functions and Applications of Medicinal Plants, Guizhou Medical University, Guiyang 550025, China; 4 Key Laboratory of Endemic and Ethnic Diseases, Ministry of Education & Key Laboratory of Medical Molecular Biology of Guizhou Province, Guizhou Medical University, Guiyang 550004, China

**Keywords:** porous hydrogels, immunomodulation, immunotherapy, cancer therapy, tissue regeneration

## Abstract

Cancer immunotherapy relies on the insight that the immune system can be used to defend against malignant cells. The aim of cancer immunotherapy is to utilize, modulate, activate, and train the immune system to amplify antitumor T-cell immunity. In parallel, the immune system response to damaged tissue is also crucial in determining the success or failure of an implant. Due to their extracellular matrix mimetics and tunable chemical or physical performance, hydrogels are promising platforms for building immunomodulatory microenvironments for realizing cancer therapy and tissue regeneration. However, submicron or nanosized pore structures within hydrogels are not favorable for modulating immune cell function, such as cell invasion, migration, and immunophenotype. In contrast, hydrogels with a porous structure not only allow for nutrient transportation and metabolite discharge but also offer more space for realizing cell function. In this review, the design strategies and influencing factors of porous hydrogels for cancer therapy and tissue regeneration are first discussed. Second, the immunomodulatory effects and therapeutic outcomes of different porous hydrogels for cancer immunotherapy and tissue regeneration are highlighted. Beyond that, this review highlights the effects of pore size on immune function and potential signal transduction. Finally, the remaining challenges and perspectives of immunomodulatory porous hydrogels are discussed.

## 1. Introduction

Immunotherapies aim to modulate the immune system to amplify innate or adaptive immunity, which is of crucial importance for the treatment of cancer and damaged tissue [1]. Cancer immunotherapies that aim to utilize, modulate, activate, and train the immune system to amplify antitumor T-cell immunity, such as monoclonal antibodies [2], adoptive cell therapies [3], oncolytic viruses [4], cytokines, and chemokines [5], are promising approaches for treating or even curing cancers. Additionally, the immune system has also attracted wide attention for tissue development and regeneration due its ability to defend against external challenges, such as modulating macrophage M2 polarization to favor tissue regeneration [6,7,8].

Hydrogels are a subgroup of biomaterials comprising covalently or noncovalently crosslinked hydrophilic polymer networks [9,10] and are promising immunomodulatory materials for cancer treatment and tissue engineering as they can replicate the structure and biological environment of the native extracellular matrix [11,12,13]. Currently, hydrogels based on immunomodulatory and tissue engineering are receiving increasing attention. For instance, Xiong et al. summarized recent studies on functional hydrogels for altering the immune microenvironment of diabetic foot ulcer, and physicochemical properties essential for the design of regenerative hydrogels for immunomodulation [14]. However, conventional hydrogels with submicron or nanosized pore structures are unfavorable for cellular infiltration and survival and thus lead to unsatisfactory treatment results [15,16]. Hydrogels with a sufficient pore size show promise for overcoming some of the current limitations of conventional hydrogels on cell behavior [17]. At present, most studies have focused on the effects of porous hydrogels on the host immune response [18,19,20]. Few reports have described how the porous structure of hydrogels affects the immune response in cancer therapy or tissue regeneration.

In this review article, we critically review the recent progress on developing immunomodulatory porous hydrogels for cancer therapy and tissue regeneration. We begin with putting forward an overview of design strategies for developing porous hydrogels while discussing their potential and their challenges. Then, the current status of the immunomodulatory effects of porous hydrogel systems for cancer therapy and tissue regeneration is reviewed. Finally, the challenges and future perspectives associated with immunomodulatory porous hydrogels are highlighted.

## 2. Strategies to Develop Porous Hydrogels

**Figure 1:**
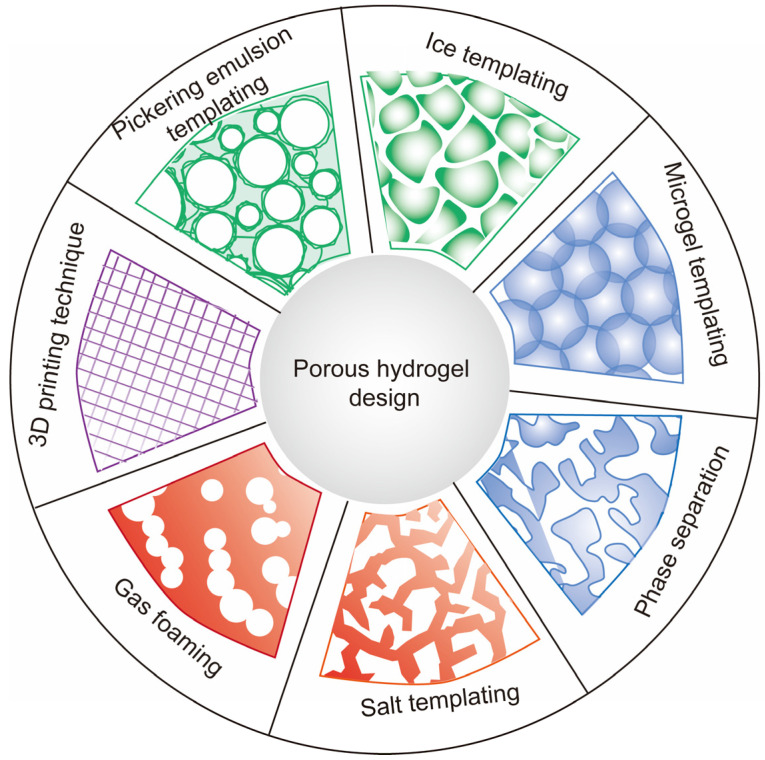
Common strategies to develop porous hydrogels.

The presence of voids in a bulk hydrogel can dramatically change its performance. If voids are not simply randomly trapped air, then the hydrogel can be defined as porous. Porous hydrogels are characterized by porosity, pore size distribution, interconnectivity of pores, and the ordered/disordered state of the pore structure [21,22]. According to pore size, porous hydrogels are divided into different classifications, such as nonporous, microporous, macroporous, and superporous hydrogels. As shown schematically in Figure 1, several approaches are available to generate porous hydrogels, mainly ice templating [23], Pickering emulsions templating [24,25], microgel templating [26], phase separation [27], salt templating [28], gas foaming [29], and 3D printing [30]. In this section, we will summarize the physical performance and preparation strategies that incorporate physical pores into hydrogels.

### 2.1. Ice Templating

**Figure 2:**
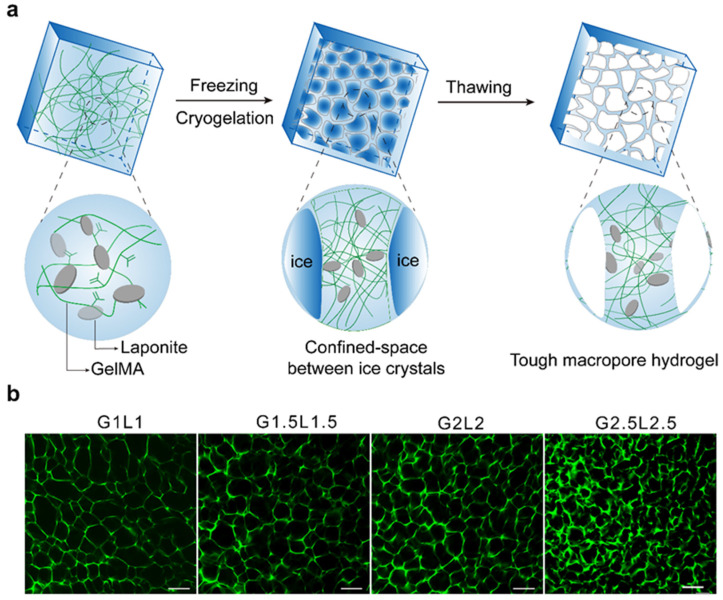
Shape-recoverable porous nanocomposite hydrogels developed using the ice templating technique. (**a**) Schematic diagram of the fabrication process for porous hydrogels. (**b**) CLSM images of porous hydrogels with different total mass concentrations; scale bar: 200 μm. Adapted with permission [33]. Copyright 2022, American Chemical Society, Washington, DC, USA.

Ice templating aims to use the crystallization of water as a pore-forming agent to develop porous hydrogels [31]. Therefore, the ice templating technique is friendly to the environment as it forms a microporous structure within the hydrogel without the need for toxic organic solvents [32]. More importantly, the mechanical performance of the obtained hydrogels is superior to those of other porous hydrogels because pore walls with covalently or noncovalently crosslinked bonds are generated during the freezing step [15,23]. In 2022, our group reported a shape-recoverable porous nanocomposite hydrogel by utilizing ice templating polymerization [33]. The porous hydrogel was developed through chemical crosslinking at −20 °C. When the water was frozen, ice crystals formed and subsequently expelled the polymer precursors, which concentrated into small semi-frozen regions. Chemical crosslinking occurred around the ice crystals, leading to a chemical crosslinked polymer network (Figure 2a). When the ice crystals thawed, an interconnected porous structure was revealed. As shown in Figure 2b, an interconnected pore structure was obtained through this method.

**Figure 3:**
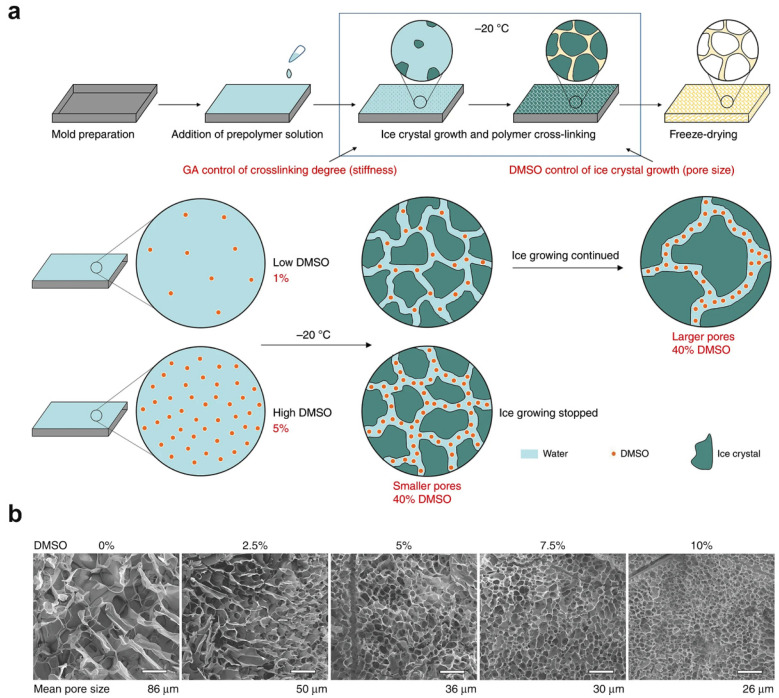
Control of hydrogel micropore size by DMSO-regulated ice crystal growth. (**a**) A controllable mechanism of ice crystal growth by adding a DMSO cryoprotectant. (**b**) SEM images of a porous gelatin hydrogel with varied DMSO cryoprotectant concentrations; scale bar: 100 μm. Adapted with permission [38]. Copyright 2019, The Author.

In addition, the microporous size and morphology can be easily manipulated by controlling ice crystal growth, mainly through inert additions of chemical ice-inhibition molecules [34], changes in the freezing temperature and rate [35], and changes in the monomer, polymer, and crosslinker concentrations [36]. Chemical ice-inhibition molecules, such as cryoprotectant and antifreeze proteins, can effectively modulate and inhibit ice crystal growth and thus are capable of producing hydrogels with different pore sizes [37]. For example, Du et al. found that cryoprotectants enabled ice crystal growth control, which was used to realize decoupling of scaffold stiffness and pore size [38]. As shown in Figure 3a, dimethyl sulfoxide (DMSO) enrichment during cryo-gelatinization can change the freezing point of a reactive polymer precursor solution, which subsequently influences the corresponding ice crystal formation. Therefore, hydrogel scaffolds with different pore sizes can be prepared by modulating the initial DMSO concentration. SEM images showed that a decrease in pore size was observed with increased DMSO concentration (Figure 3b). Apart from DMSO, Shoichet et al. reported the possible feasibility of adding biocompatible carbohydrate cryoprotectants into a hyaluronic acid precursor solution, as these sugars interact with water to subsequently modulate ice crystal growth, leading to transparent porous hydrogels with different pore sizes [39].

**Figure 4:**
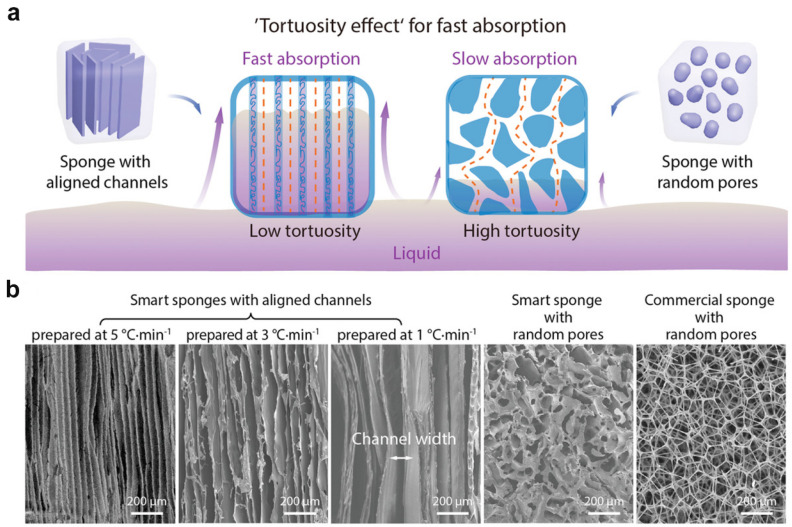
Control of hydrogel micropore morphology by modulating ice crystal growth directionally along the temperature gradient. (**a**) Schematic diagram of a porous scaffold with aligned channels and random pores. (**b**) SEM images of representative porous structures under various freezing conditions. Adapted with permission [40]. Copyright 2020, Wiley−VCH, Weinheim, Germany.

In addition to ice inhibition molecules, freezing conditions can also modulate ice crystal growth, thus leading to porous structures. Recently, Bai et al. reported a porous polyurethane-based scaffold with an aligned channel structure [40]. As shown in Figure 4a, the freezing speed modulated the porous structure during the freezing process. SEM images indicated that more ice crystals nucleated at a higher cooling rate, leading to more channels but with a smaller channel width (Figure 4b). The average channel width decreased from 155 to 50 µm when the cooling rate increase from 1 to 5 °C/min. With respect to ice templating strategies, they retain certain advantages over other templating techniques, such as realizing pore interconnectivity and template removal without extra processing steps. Although freezing speed rates and cryoprotectants can be utilized to regulate pore size distribution, there remains many challenges for porous hydrogels based on the ice templating technique, such as a broad pore size distribution and minimal directionality.

### 2.2. Pickering Emulsion Templating

Emulsions are composed of two immiscible liquid phases in which the dispersed phase is decentralized in a continuous phase as microscopic or colloidal drops. Emulsions are thermodynamically unstable but can be dynamically stable in the presence of an emulsifier [41,42]. Surfactant or colloidal particle-stabilized macroemulsions are termed as Pickering emulsions. If the volume fraction of the internal phase is noticeably greater than 0.74, the emulsions are considered high internal phase Pickering emulsions (HIPPEs) [43,44,45]. If the continuous aqueous phases contain polymerizable monomers or hydrogel precursors and crosslinkers, HIPEs and HIPPEs can undergo concentrated emulsion templating to develop porous hydrogel with subsequent polymerization or gelatinization [46].

**Figure 5:**
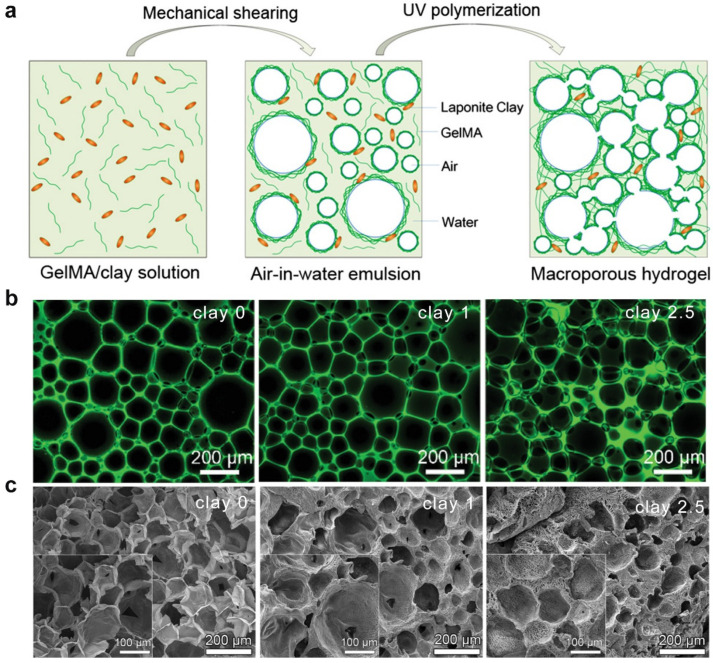
Gelatin methacryloyl-stabilized air-in-water emulsion templating for porous nanocomposite hydrogels. (**a**) Schematic diagram of the porous nanocomposite hydrogels based on gelatin methacryloyl-stabilized emulsion templating. (**b**) CLSM images of gelatin methacryloyl-stabilized air-in-water emulsion templating. (**c**) SEM images of porous nanocomposite hydrogels with varied amounts of clay. Adapted with permission [48]. Copyright 2020, Wiley-VCH.

In 2017, Chen et al. reported HIPEs prepared from a supramolecular cellulose nanocrystal stabilizer via one-step emulsification, which was used as a template for developing interconnected porous hybrid composite hydrogels [47]. In this contribution, a quadruple hydrogen bond moiety, 2-ureido-4[1H]-pyrimidone (UPy)-modified cellulose nanocrystal (CNC), was first developed through simple free radical polymerization. Next, the prepared CNC-UPy-stabilized HIPEs were utilized as templates, and a co-polymerization of the aqueous phase containing acrylamide and gelatin methacrylate monomers enabled the formation of cell-adhesive porous hybrid composite hydrogels. However, the oil phase and impurities needed to be removed by thoroughly washing with ethanol and water. Since then, the group has developed a gelatin methacryloyl stabilized air-in-water emulsion, and highly porous hydrogels were developed by utilizing a concentrated emulsion template [48,49,50]. As shown in Figure 5a, porous nanocomposite hydrogels were developed by using gelatin methacryloyl-stabilized air-in-water emulsions, which did not require organic solvents, purification steps, or extra surfactants. The average droplet size decreased by increasing the amount of clay (Figure 5b). Subsequently, porous hydrogels were developed through co-polymerizing gelatin methacryloyl with acrylamide, in which the surface roughness of the pore is dependent on the clay concentration (Figure 5c).

**Figure 6:**
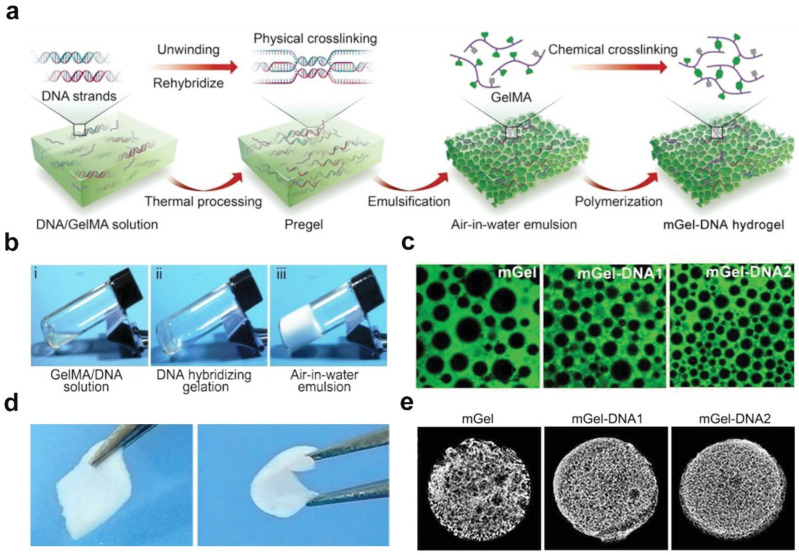
Double-network DNA porous hydrogels via the emulsion template technique. (**a**) Schematic illustration of designing double-network DNA porous hydrogels via the emulsion template technique. (**b**) Photographs of emulsion templates formed by gelatin methacryloyl and a DNA precursor solution. (**c**) CLSM images of gelatin methacryloyl- and DNA solution-stabilized air-in-water emulsion droplets. (**d**) Photographs of a porous hydrogel before and after bending with tweezers. (**e**) Micro-CT images of a porous hydrogel with different gelatin methacryloyl and DNA solution concentrations. (**a**–**e**) Adapted with permission [51]. Copyright 2023, Wiley-VCH.

Recently, they reported a double-network DNA porous hydrogel via air-in-water emulsion templating (Figure 6a), in which the physical self-assembly of DNA strands and covalently crosslinked gelatin chains enabled the formation of a deformable double network [51]. As shown in Figure 6b, gelatin methacryloyl with 80% substituent-stabilized air-in-water emulsion droplets remained stable after exposure to a high-speed shearing force, in which the composite emulsion size decreased from 50 μm to 150 μm, forming an emulsion template that was used to develop functional porous hydrogels (Figure 6c). The formed porous hydrogels showed good shape recovery performance (Figure 6d) and highly interconnective porous structures (Figure 6e). The advantages of Pickering emulsion templating include easy processing, scalability, and good pore interconnectivity. Nevertheless, porous hydrogels prepared from Pickering emulsion templating usually lack a certain topography due to their limited architecture and resolution. The orientation of the pores is also difficult or impossible to control. Recently, the precise characteristics of 3D printing techniques effectively circumvented the disadvantages of Pickering emulsions.

### 2.3. Microgel Templating

**Figure 7:**
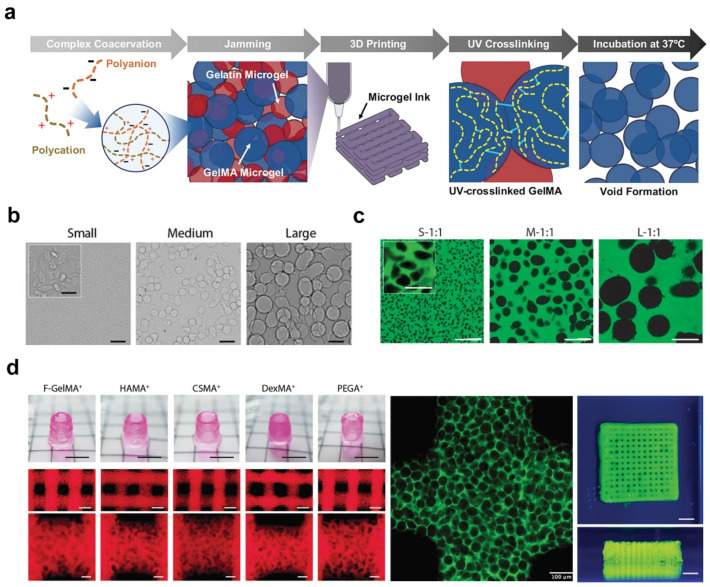
Tunable sacrificial gelatin microgel templating for 3D bioprinted hydrogels. (**a**) Schematic diagram depicting the process for producing porous hydrogels by sacrificial microgel templating. Adapted with permission [56]. Copyright 2021, Wiley−VCH. Representative brightfield (**b**) and CLSM images (**c**) of gelatin microgel templates in different size ranges. (**d**) Representative photograph of a printed lattice and tubular structure with different matrix materials. Scale bars: (**b**,**c**) 100 μm; (**d**) (top left/right panel) 5 mm; (**d**) (middle left panel) 500 μm; (**d**) (bottom left panel/middle panel) 100 μm. Adapted with permission [54]. Copyright 2022, Wiley−VCH.

Microgel biomaterials comprise packed solid microgels, and controlling the microgel size and concentration can also produce porous hydrogels [52,53,54]. For example, gelatin was dissolved in a warm water-ethanol mixture supplemented with pluronic F127 and gum arabic surfactants to form a miscible phase. When the temperature of the mixture was decreased to room temperature, the gelatin phase separated from the mixture and formed a spherical microgel because of its poor solubility in ethanol and thermal gelatinization in water. Therefore, gelatin microgels can be used as sacrificial templates, leading to porous hydrogels [55]. As shown in Figure 7a, Heilshorn et al. proposed a strategy to develop 3D printing of microgel scaffolds using sacrificial microgel templates that consisted of gelatin and gelatin methacryloyl. After printing and light-induced covalent crosslinking, the sacrificial microgels were readily removed by incubation at 37 °C [56]. Similarly, Stevens et al. also reported a gelatin-based microgel-templated porogel bioink platform to develop 3D bioprinted hydrogels with controlled microporosity. As shown in Figure 7b,c, small, medium, and large-sized microgels were generated. Brightfield and CLSM images revealed micropores with diameters from 10 μm to 100 μm. All the tested microgel-templated porogel bioinks were based on different natural methacryloyl polymers, such as gelatin, hyaluronic acid, chitosan, and dextran, which all exhibited excellent printability with both lattice and tubular structures (Figure 7d).

**Figure 8:**
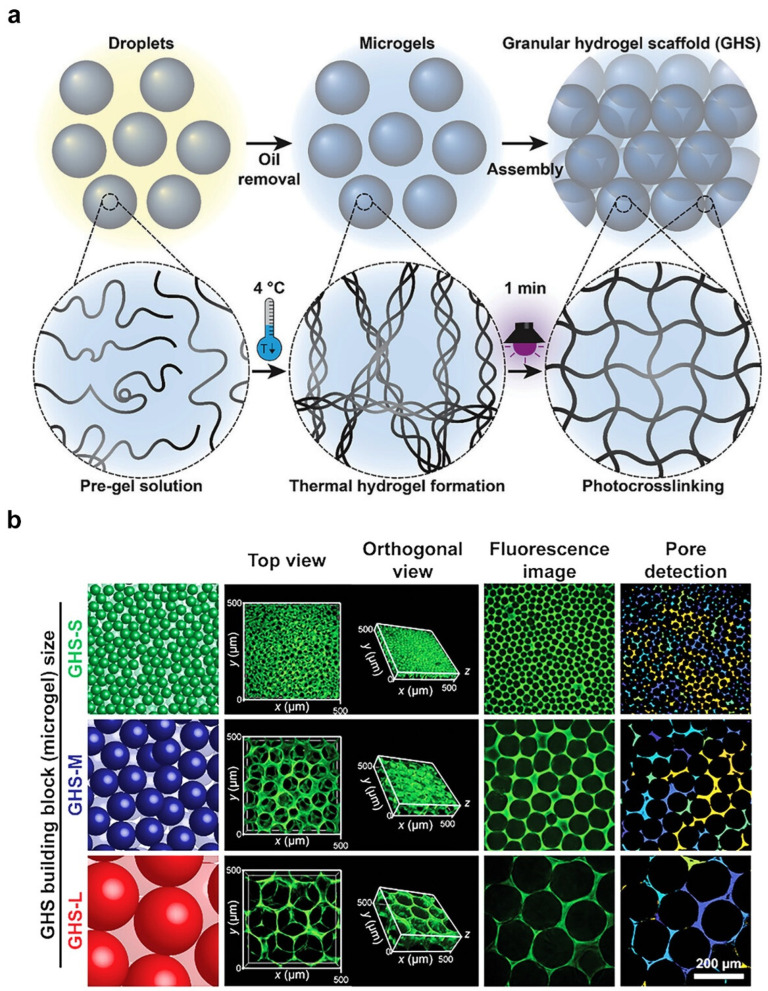
Fabrication of porous hydrogels via microgel templating. (**a**) Microgels were formed by a physically crosslinked gelatin methacryloyl precursor solution. (**b**) Porous hydrogels with a tunable pore size. Adapted with permission [57]. Copyright 2023, Wiley-VCH.

Sheikhi et al. utilized a granular hydrogel scaffold (GHS) technique to develop pore-forming hydrogels, which used three microgels of different sizes to precisely control the pore architecture of the hydrogel [57]. As shown in Figure 8a, gelatin methacryloyl droplets were first converted to microgels via physical crosslinking at 4 °C, and subsequently, they formed a GHS by photo-crosslinking–induced packing and chemical assembly. As shown in Figure 8b, GHSs with a tunable pore size were developed using different microgels. Small, medium, and large microgels generated GHSs with pore diameters of 12, 20, and 44 µm, respectively. The merit of microgel templating is that the specific dimensions of the pore can be realized. However, microgels are not appropriate in applications in which full pore interconnectivity is desired, because statistically, some fractions of the pores are not fully continuous [58]. Microgel templating uses complex extraction steps, particularly if the application requires the template to be completely removed to regulate cell behavior.

### 2.4. Phase Separation

**Figure 9:**
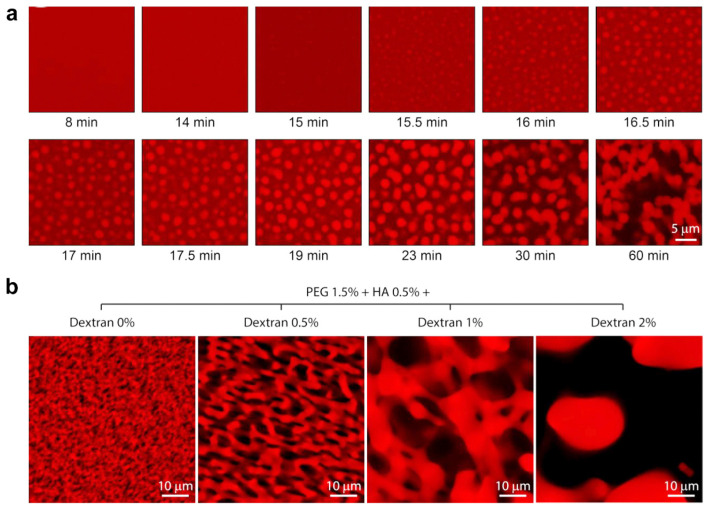
Porous hydrogels derived from aqueous dynamic phase separation. (**a**) Monitoring of the phase separation in the process of gelatinization. (**b**) Controllable pore size with various dextran concentrations. Adapted with permission [63]. Copyright 2019, Elsevier, Amsterdam, The Netherlands.

Phase separation is the aggregation of different types of molecules into two or more apparently immiscible phases in a mixture under specific conditions [59,60,61]. In each phase, the noncovalent forces between the molecules cause them to clump together, forming spherical structures or droplets. These can exist stably because a specific molecule is spatially isolated from the surrounding environment and can be used to develop porous hydrogels [62]. For example, Zenobi-Wong et al. presented an interconnected porous network based on phase separation, in which pure hyaluronic acid was used for polyethylene glycol (PEG) exclusion, and then different dextran concentrations were introduced to control pore size. Exclusion of PEG from the hyaluronan phase improved the click chemistry crosslinking kinetics. Additionally, the phase separation between hyaluronan and PEG also led to a more stable hydrogel network compared to that of the bulk hydrogel [63]. As shown in Figure 9a, phase separation occurred through chain elongation of PEG during the crosslinking reaction, and the high viscosity prevented the phases from decanting or collapsing into microspheres when the porous hydrogel had not yet formed. In addition, porous hydrogels with a tunable pore size are generated by changing the dextran concentration (Figure 9b). The phase separation enables the development of porous hydrogels with open and interconnected pore structure [64]. The porosity of hydrogels can be controlled by manipulating the phase separation degree. However, the pore-forming hydrogels prepared from phase separation usually exhibit poor mechanical performance.

### 2.5. Salt Templating

**Figure 10:**
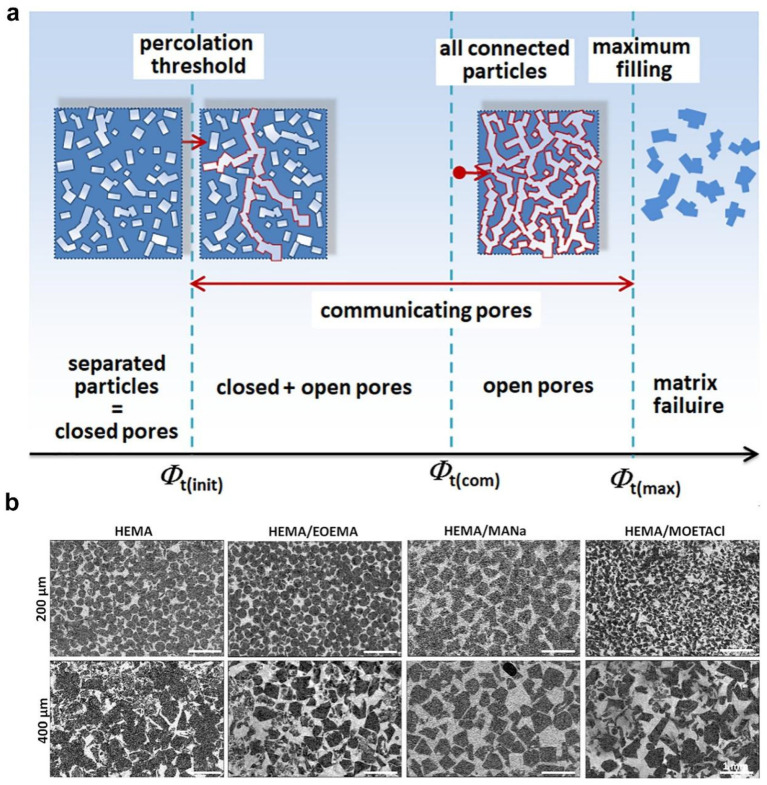
PHEMA-based porous hydrogels prepared with NaCl particle templating. (**a**) Pore-forming hydrogel volumes generated by the NaCl salt templating technique. (**b**) Cross-section illustrations of PHEMA-based porous hydrogels. Adapted under the terms of the CC-BY Creative Commons Attribution 4.0 International license [66].

Salt templating is another common approach for developing porous hydrogels due to its simple operation and low cost. Salt crystals are introduced into polymer precursor solutions, and porous hydrogels are formed through a covalently or noncovalently crosslinked polymer network with removal of the salt crystal template [65]. NaCl salt crystals are a commonly used salt crystal template because of their availability and bioinertness. For example, Kubies et al. utilized an NaCl crystal template to develop porous hydrogels with interconnected porous structures in which sequences of embedded packed salt particles with arbitrary sizes were used [66]. As shown in Figure 10a,b, porous hydrogels were prepared based on the salt templating technique, in which the morphology of the NaCl crystal templates was dependent on the matrix composition. The major weakness of the salt templating technique is the high osmolarity, resulting from a high salt concentration. Additionally, there is still a risk that salt crystals may remain within the porous hydrogel.

### 2.6. Gas Foaming

**Figure 11:**
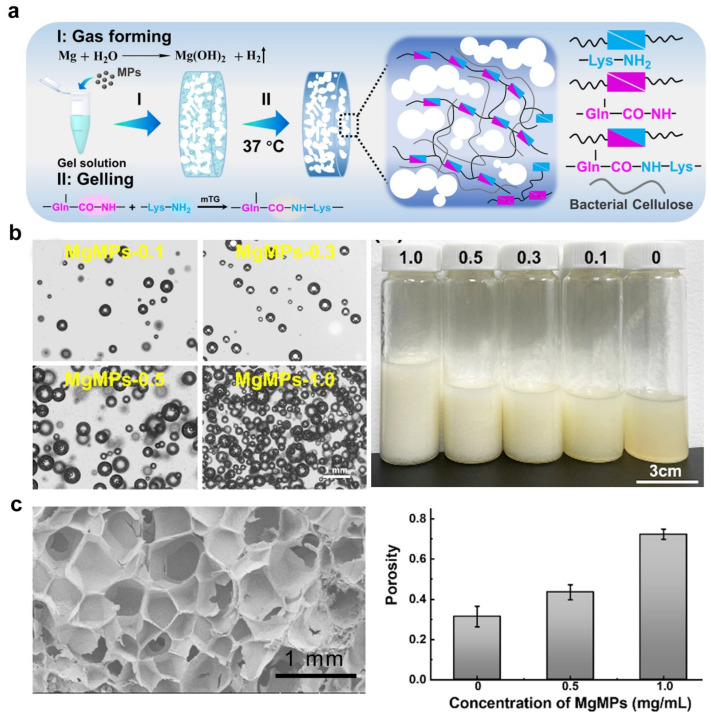
Injectable porous hydrogel templated by a gasification reaction. (**a**) Schematic illustration depicting the gas templating for a porous hydrogel. (**b**) Microscope images and photograph of hydrogels loaded with varied Mg microparticles. (**c**) SEM images and porosity of the porous hydrogels. Adapted under the terms of the CC-BY Creative Commons Attribution 4.0 International license [69].

The principles of gas foaming include in situ-generated gas bubbles in the process of polymer precursor solution gelatinization, leading to the formation of a pore-forming hydrogel [67]. The most frequently used gas foaming agents are carbonates and nitrites, which have negative effects on encapsulated cells; thus, it is of great significance to form spontaneous bubbles within the cell-laden hydrogel. Recently, the concept of in situ void formation was put forward to develop cell-friendly porous hydrogels, using a nontoxic porogen and hydrogel precursor solution, followed by in situ formation of voids [21]. Mg particles are appealing as degradable foaming agent templates for preparing porous hydrogels because Mg can be corroded in vivo, simultaneously generating hydrogen gas. In particular, soluble magnesium ions enable continuous hydrogen release when Mg particles degrade, which favors cell proliferation to realize angiogenesis and osteogenesis [16]. For example, Jiang et al. reported a cost-effective method to develop porous hydrogels by in situ gas foaming [68]. In 2023, Wang et al. reported an in situ gas-forming approach for developing porous hydrogels with Mg microparticles, in which the gasification reaction of magnesium in water occurred [69]. As shown in Figure 11a, the hydrogen bubbles derived from the Mg microparticle reaction in water led to a porous hydrogel. Microscope images showed that the number of gas bubbles depended on the Mg microparticle concentration (Figure 11b). Large interconnected pores within the hydrogel were observed, indicating that the porosity was associated with the Mg microparticle concentration (Figure 11c). Although gas foaming is low cost, it lacks a controllable interconnected and directional porous structure. Additionally, it is difficult to remove the extracted residues resulting from the solid gas foaming agents. Furthermore, the process of gas foaming is random, which is unfavorable for developing hydrogels with a uniform pore structure.

### 2.7. Three-Dimensional Printing Technique

**Figure 12:**
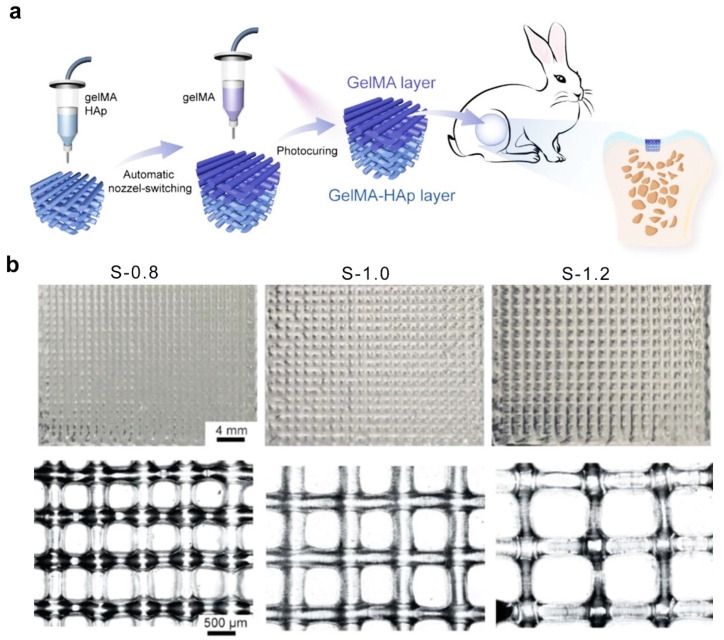
Porous hydrogel prepared via 3D printing. (**a**) Schematic diagram of bi-layered porous hydrogel generation via 3D printing. (**b**) Top view and SEM images of porous hydrogels with different interfilament spacings. (**a**,**b**) Adapted with permission [74]. Copyright 2020, Wiley-VCH.

3D printing is also a method for the development of hydrogels with internal pore structures as well as personalized shapes [67]. In this technique, the porous hydrogel structure is rendered on a computer and printed layer-by-layer into the desired porous hydrogel. More generally, according to the gelation chemistry, the mechanisms by which the porous structure are produced are divided into templated and free form [70]. With respect to templated systems, the polymer precursor solution is first deposited around a pre-printed sacrificial template or printed as a liquid that is subsequently polymerized in situ, and then removal of the sacrificial template leads to the desired microporous structure. However, current printing strategies still suffer from the removal of a porogen or unpolymerized monomer following gelation [67]. Free-form printing techniques offer a partial alternative to directly printing a porous structure. The gelatinization mechanisms mainly include physical or chemical crosslinking, such as UV-induced free radical polymerization [71], electrostatic interactions [72], and enzyme-mediated crosslinking [73]. For example, Ding et al. created photo-crosslinked bilayered porous hydrogel scaffolds by using free-form printing techniques [74]. As shown in Figure 12a, the bilayered porous hydrogel scaffolds were developed through continuous 3D printing. The diameter of the nozzles was about 0.21 mm. The center distance of the printed filament was preset to be 0.8, 1.0, or 1.2 mm, and the corresponding samples were designated as S-0.8, S-1.0, and S-1.2, respectively (Figure 12b). The advantage of the 3D printing technique is that it can fabricate complex microstructures with a well-matched mechanical performance. Currently, the 3D printed scaffold not only has bio-adhesive properties to provide support for cells, but also has the potential for shape memory to contract synchronously with tissues [75,76]. However, 3D printing is limited by the choice of bio-ink, which requires an appropriate viscoelasticity. In addition, porous hydrogels prepared by 3D printing are on the millimeter or centimeter scale, which is not sufficient to develop micron-scale structures.

## 3. Porous Hydrogel-Assisted Immunomodulation for Cancer Therapy

**Figure 13:**
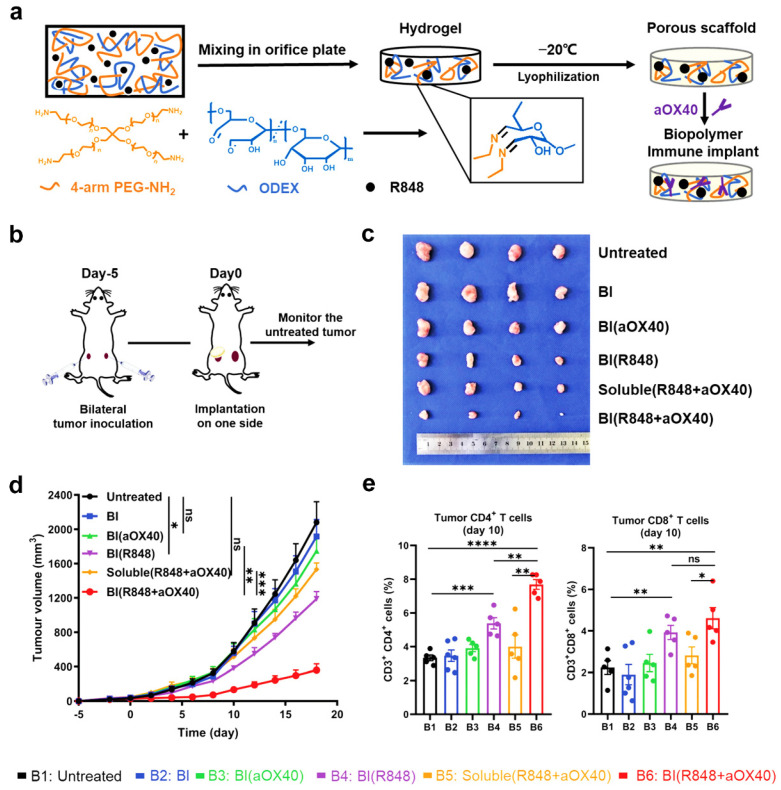
Sequential activation of innate and adaptive immunity for distal tumor therapy based on a porous hydrogel. (**a**) Schematic diagram of the development of an implantable porous hydrogel. (**b**) Schematic diagram of bilateral tumor inoculation. (**c**) Photograph of resected distal tumors. (**d**) Growth curves of distal tumors. (**e**) CD4+ and CD8+ T cells in the tumors. (* *p* < 0.05, ** *p* < 0.01, *** *p* < 0.001, **** *p* < 0.0001 and ns = no significance). Adapted with permission [87]. Copyright 2020, Wiley−VCH.

Cancer immunotherapy relies on the insight that the immune system can be used to defend against malignant cells. The aim of cancer immunotherapy is to utilize, modulate, activate, and train the immune system to amplify antitumor T-cell immunity [77,78]. Hydrogel systems are favorable for the delivery of therapeutic agents or cells for cancer therapy. More importantly, hydrogels can be designed with appropriate biochemical or biophysical cues for recruiting endogenous immune cells and can regulate their functions to improve the tumor microenvironment [79,80]. Additionally, porous hydrogels with high porosity and large pores can provide sufficient space for cell migration and growth, facilitate nutrient and metabolite exchange, and promote cell-to-cell communication [81,82]. In addition, porous hydrogels are soft and injectable, providing the advantages of non-invasive use in surgical procedures [83,84]. Hence, the use of immune-modulating biomaterials constructed upon porous scaffolds has the potential to elicit more robust anti-tumor immune responses, thereby fostering the advancement of cancer immunotherapy [85,86]. For example, Chen et al. developed a porous biopolymer immune implant for postoperative treatment of colorectal cancer, which was characterized by tissue adhesion, sustained drug release, and immune memory [80]. As shown in Figure 13a, porous hydrogels were prepared by a Schiff base reaction crosslinking 4-arm PEG amine and oxidized dextran, and then resiquimod and anti-OX40 antibody were encapsulated within the porous hydrogel. Subsequently, the drug-loaded porous hydrogels were injected into a tumor in situ (Figure 13b). As shown in Figure 13c,d, the drug-loaded porous hydrogel exhibited better anti-tumor effects compared with the drug-free hydrogel. More importantly, the porous drug-loaded group recruited a large number of CD8+ and CD4+ T cells, enhancing the anti-tumor immune response in situ (Figure 13e).

**Figure 14:**
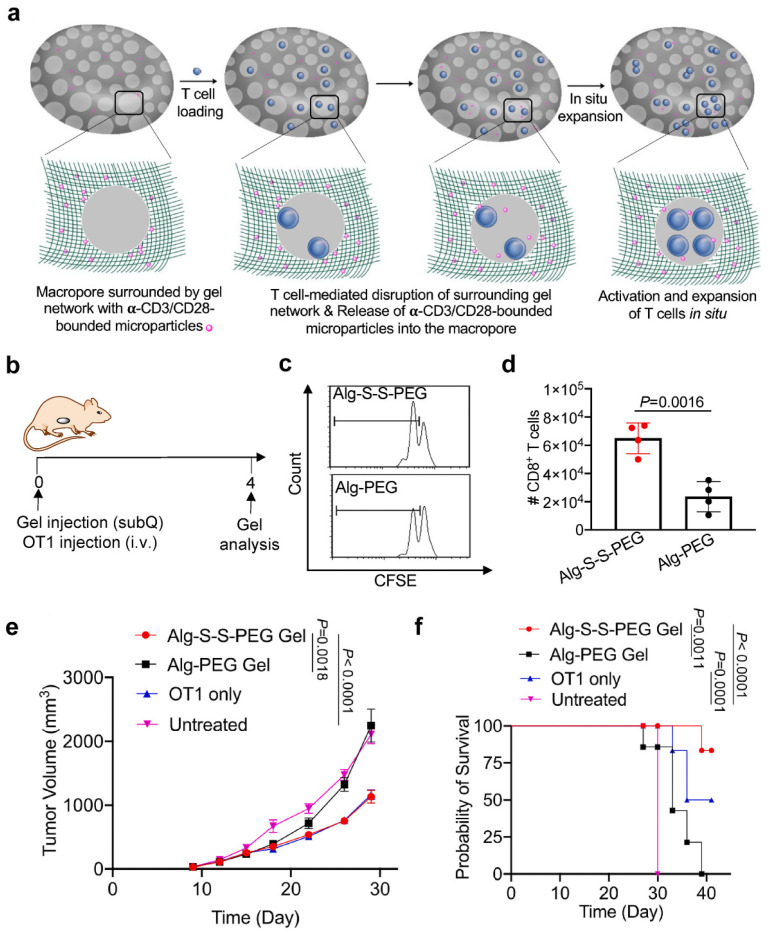
T-cell–responsive porous hydrogels for in situ T-cell expansion for cancer therapy. (**a**) Schematic diagram for developing T-cell–responsive porous hydrogels. (**b**) Schematic diagram of the time frame of this study. (**c**) Representative histograms of CFSE-stained OT-1 cells within the porous hydrogel. (**d**) Total number of CD8+ T cells within the porous hydrogel. (**e**) Average tumor volume in each group. (**f**) Kaplan–Meier plot of all the groups. Adapted with permission [88]. Copyright 2023, Elsevier.

In addition to serving as a carrier for immunoadjuvants, porous hydrogels can also serve as a carrier for realizing tumor T-cell expansion and release in situ. In 2022, Wang et al. reported a porous hydrogel that exhibited T-cell responsiveness, facilitating T-cell expansion in situ and amplifying the antitumor immune response, in which 150-µm macropore structures were formed, which then accommodated T cells in situ [81]. As shown in Figure 14a, the prepared porous hydrogel enabled the controllable release of α-CD3a/CD28-bound microparticles, subsequently activating and expanding T cells in situ (Figure 14b). At 4 days post-implantation, the number of CD8+ T cells in the Alg-S-S-PEG porous hydrogel was higher than that in the Alg-PEG hydrogel, indicating that the porous structure favored expanding T cells in situ (Figure 14c,d). In a therapeutic setting, the porous hydrogel group also demonstrated significant inhibition of tumor growth and prolonged survival (Figure 14e,f).

Currently, it is widely acknowledged that tumor immunotherapy faces numerous challenges, including immune suppression within the tumor microenvironment, limited functionality of T lymphocytes, and inadequate trafficking and infiltration, all of which hinder the successful initiation of anti-tumor effects. In summary, porous hydrogels have the potential to facilitate simultaneous delivery and release of cells and drugs, on-site activation and transportation of immune cells, and recruitment of immune cells, thereby augmenting the anti-tumor immune response. Therefore, the exploitation of immunotherapeutic biomaterials based on porous materials is expected to become an effective strategy to enhance tumor immunotherapy.

## 4. Porous Hydrogel-Assisted Immunomodulation for Tissue Regeneration

One way to achieve in situ regeneration of damaged tissue is by developing hydrogel-based cell delivery systems for implantation [89,90]. Beyond that, in situ tissue regeneration can also be realized by manipulating biochemical and biophysical cues to recruit endogenous cells through engineered functional tissues [9,91]. There is growing recognition that the biophysical cues of hydrogels, such as stiffness, viscoelasticity, pore size, and porosity, also significantly impact cell responses, thus leading to final regenerative outcomes [92,93]. In addition to mechanical performance, pore characteristics such as pore size and pore distribution within the hydrogel are also considerable concerns for final tissue regeneration [17,94]. Hydrogels with porous structures beyond the mesh size show greater benefits for nutrient transportation and metabolite discharge, cell infiltration, and tissue formation in situ compared to conventional hydrogels [21]. Recently, many hydrogels with a tunable pore size have been defined as key aids for cell guidance, regulation, and final tissue regeneration, such as in vascular remodeling [95], nerve regeneration [63], healing of infected deep burn wounds [96], and vascularized bone regeneration [68]. However, implanting these porous hydrogels into the body is challenging, as the host recognizes most implants as foreign objects, and complex signaling cascades occur in the process of tissue regeneration, which determine the success or failure of the implant [97]. Thus, it is of utmost importance to understand the pore structure parameters of hydrogels, such as pore size and porosity, that can induce a pro-inflammatory or anti-inflammatory immune response [98].

In addition to their early recognition of pathogens and phagocytic function in host defenses, macrophages are one of the main immune cells that participate in organ development, homeostasis, and regeneration [99,100]. According to phenotypic and functional diversity, macrophages are divided into diverse phenotypes between two extremes, the classically activated M1 type and the alternatively activated M2 type [101]. Macrophage polarization is associated with the release of growth factors and cytokines from the adjacent microenvironment. M1 macrophages, which are commonly induced by lipopolysaccharide (LPS) and interferon γ (IFN-γ), predominately secrete pro-inflammatory cytokines, such as tumor necrosis factor-α (TNFα) and interleukin (IL)-1b and IL-6. However, M2 macrophages, which are often stimulated by IL-13 and IL-4, predominately secrete anti-inflammatory cytokines, such as IL-10 and arginase-1 (ARG-1) [101].

**Figure 15:**
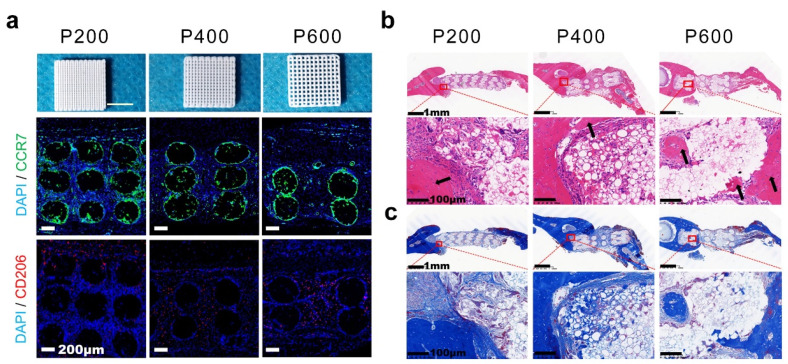
The pore size of 3D-printed scaffolds promotes bone regeneration by regulating macrophage M2 polarization. (**a**) Image of immunofluorescence staining showing the scaffolds with different pore sizes for modulating the macrophage phenotype. Images of H&E (**b**) and Masson’s trichrome staining (**c**) of porous hydrogels for bone regeneration. Adapted with permission [103]. Copyright 2022, American Chemical Society.

The pore size within implanted hydrogel scaffolds modulates macrophage polarization and provides a pro-regenerative immune microenvironment for realizing tissue regeneration [38]. For example, a collagen and chitosan hydrogel with large pores showed sustained recruitment and phenotype modulation of macrophages for improving angiogenesis and vascularization [102]. For example, Zeng et al. developed a biocompatible porous hydrogel scaffold with different pore sizes using a 3D-printing technique, and the formed porous scaffolds regulated macrophage polarization and subsequently promoted bone regeneration [103]. As shown in Figure 15a, a well-defined macrostructure and uniform pore size were observed. P600 scaffolds showed minimal M1 macrophage infiltration, and the M2/M1 ratio of the P600 scaffold group was significantly higher than that of the other groups. Additionally, the highest collagen deposition and significantly improved neovascularization were observed in the P600 group (Figure 15b,c).

**Figure 16:**
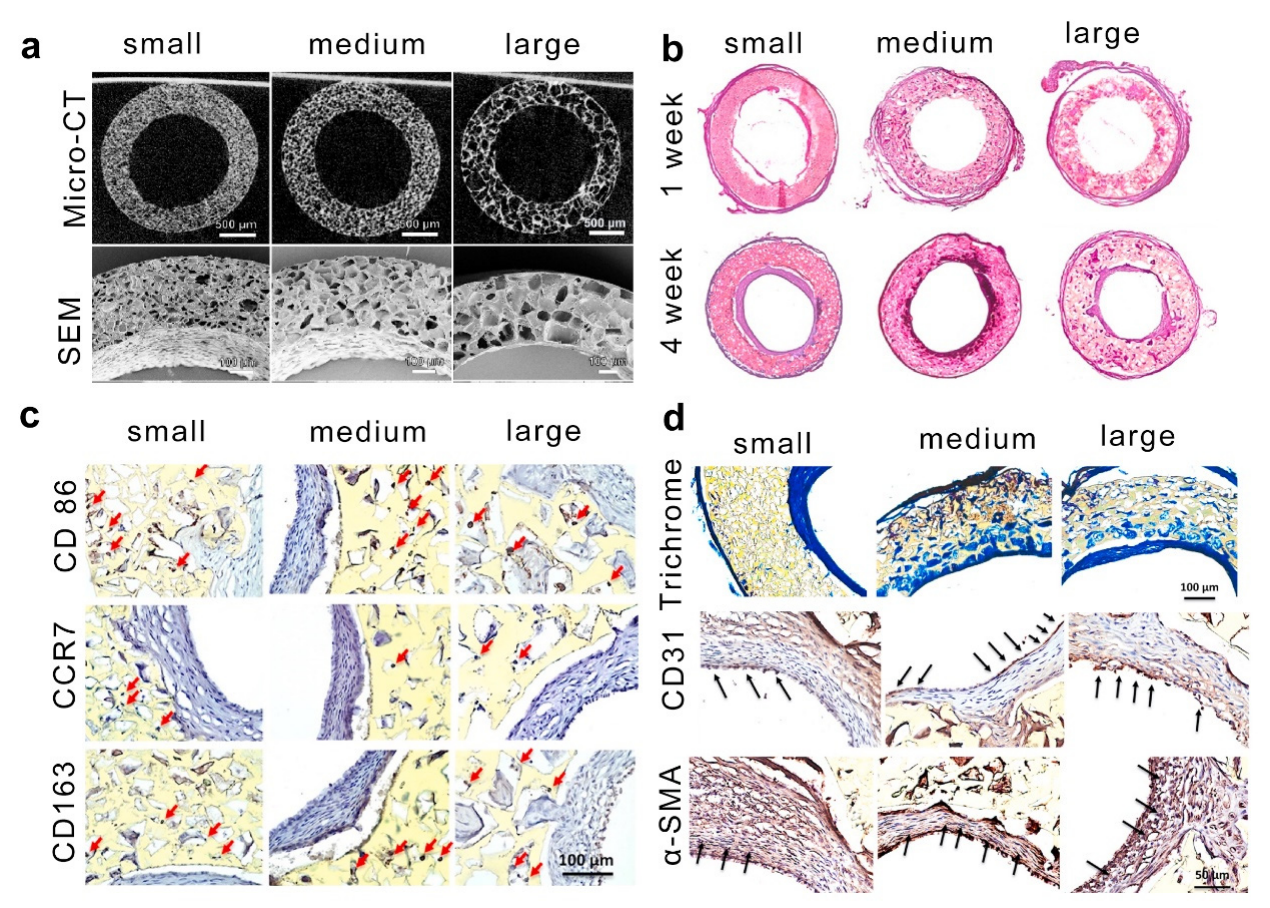
The pore size of compliant vascular grafts modulates macrophage polarization for vascular remodeling. (**a**) Micro-CT and SEM images of the graft microstructure with different pore sizes. (**b**) H&E staining of the cross-sections of the grafts with different pore sizes at various time points after implantation. (**c**) Inflammatory marker staining of the grafts with different pore sizes. (**d**) The grafts with different pore sizes regulated CD31 and α-SMA expression 4 weeks after implantation. (**a**–**d**) Adapted under the terms of the CC-BY Creative Commons Attribution 4.0 International license [105].

In addition to bone regeneration, the size of pores within implanted scaffolds was shown to play a crucial role in promoting macrophage populations with a pro-regenerative phenotype and improving vascular remodeling [104]. Recently, Yang et al. reported a bioactive porous vascular graft that modulated macrophage polarization and realized robust vascular remodeling [103]. As shown in Figure 16a, micro-CT and SEM images confirmed that a salt leaching process accompanied by the removal of porogen led to an interconnected pore structure and porous scaffolds with various pore sizes. Additionally, a rat carotid interposition model was used to investigate the ability of porous scaffolds to realize improved vascular remodeling. H&E staining results revealed neo-tissue formation in all three graft groups 4 weeks after implantation (Figure 16b). More importantly, the pore size of scaffolds regulated the macrophage phenotype, which further controlled the angiogenesis and vascularization processes (Figure 16c,d). Staining of inflammatory-related markers showed that CD86^+^, CCR7^+^, and CD163^+^ cells were all present in all three graft groups, but the macrophage polarization was different at various time points after implantation. For example, the percentage of CD163^+^ cells in grafts with medium and large pores was significantly higher than that in the small pore counterpart, indicating that the pore sizes within grafts controlled tissue remodeling outcomes.

**Figure 17:**
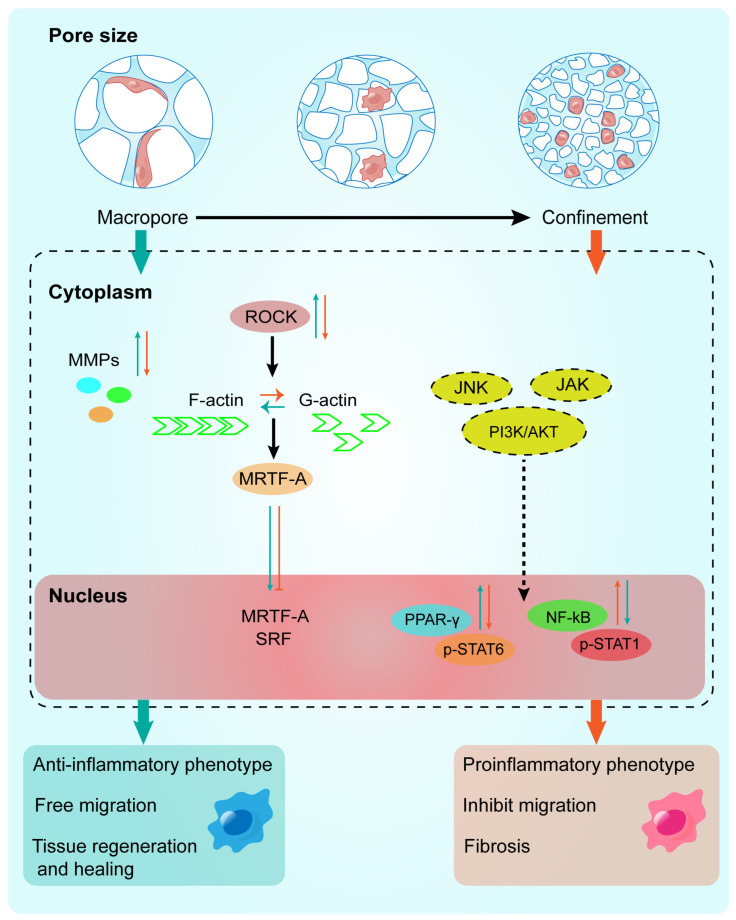
The effects of pore size on immune function and potential signal transduction. As demonstrated, macrophages cultured within the macropore structure can spread freely, exhibiting an anti-inflammatory phenotype. Macrophages cultured within the small pore structure are restricted, showing a pro-inflammatory phenotype. As macrophages extend, G-actin converts to F-actin, promoting the nuclear translocation of MRTF-A; conversely, when macrophages are restricted, the opposite occurs. Furthermore, MMPs, PPAR-γ/p-STAT6, and NF-κB/p-STAT1 signaling pathways are also regulated by pore size (dashed lines indicate predicted signal transduction). Ultimately, different pore sizes induce macrophages to exhibit distinct immune functions, leading to different disease states.

While many reports underlined the crucial role of pore size within hydrogels, how the pore size of a hydrogel influences macrophage polarization, such as the detailed mechanisms that tune macrophage polarization, differentiation, and functional plasticity, remains elusive [106,107]. It is well known that that STAT1 and STAT3/STAT6 play important regulatory roles in macrophage polarization. Liu et al. revealed that sodium alginate hydrogels with large pores activated PPARγ/STAT6 to promote M2 polarization. In contrast, the physical confinement of small pores prevented STAT6 from competing with STAT1/NF-κB for DNA binding, thereby inhibiting the expression of M2-related molecules, indicating that STAT members may play an important role in macrophage polarization induced by the pore size of hydrogels. Additionally, the critical role of the cytoskeleton in macrophage polarization also deserves special attention. Vogel et al. showed that small pores inhibited macrophage spreading and suppressed the conversion of G-actin to F-actin [106,108]. Free G-actin can bind to MRTF-A in the cytoplasm, thus affecting MRTF-A/SRF nuclear transcription and inhibiting late LPS-activated transcriptional programs during M1 activation (Figure 17). Meanwhile, small pores can restrict nuclear height, increase chromatin compaction, and limit HDAC3-mediated regulation of M1 late activation.

## 5. Concluding Remarks of Porous Hydrogel for Immunomodulatory Applications

Therapeutics targeting innate or adaptive immune systems provide a new set of tools for effective cancer treatment and tissue regeneration. Owing to their ECM-mimetic performance and multifarious bioactivities, hydrogels are a promising platform for circumventing the issues associated with systemic immunotherapy. In this review article, we reviewed the recent progress on developing immunoregulatory porous hydrogels for the treatment of cancer and damaged tissue. With respect to the strategies targeting the immune system for cancer therapy, porous hydrogel-delivered immunomodulatory agents and pore-forming hydrogel-encapsulated cells were discussed. In parallel, immunomodulation associated with the intrinsic pore structure of hydrogels was reviewed in the tissue regeneration field. The hydrogel design strategies include ice templating, Pickering emulsions templating, microgel templating, phase separation, salt templating, gas foaming, and 3D printing (Table 1). However, porous hydrogels with uniform pore size and high interconnectivity are still desired.

## 6. Future Developments of Porous Hydrogel for Immunomodulatory Applications

Despite many successful examples of immunoregulatory porous hydrogels for treatment of cancer and damaged tissue, many difficulties remain before final clinical trials and eventual approval. First, although porous hydrogels can be designed to realize controlled release of immunomodulatory agents, significant challenges remain. For example, precise spatiotemporal controllable release profiles of these immunomodulatory agents are highly desired. Additionally, porous hydrogels can also be designed to deliver therapeutic cells for local treatments of cancer. However, how therapeutic cells sense and respond to the porous structure of hydrogels is generally an overlooked area. In the tissue regeneration field, many efforts have been made to develop hydrogels with different pore structures that modulate the macrophage M1-to-M2 transition and subsequent tissue regeneration. Despite the broad applications, systematic mechanobiological investigations of three-dimensional confinement have yet to be realized because of a lack of methodologies for decoupling the scaffold stiffness and pore size.

With respect to cancer immunotherapy and tissue regeneration, the final objectives of immunomodulation are mutually contradictory. Cancer immunotherapy is devoted to building a pro-inflammatory microenvironment to amplify antitumor immune response, while a pro-regenerative microenvironment is desired for tissue regeneration. Thus, a comprehensive understanding of the role of the immune system in cancer and tissue regeneration is beneficial for developing efficient therapeutic treatments. Furthermore, interdisciplinary knowledge related to the immune microenvironment, motility, and immune regulatory functions of immune cells from the view-point of biophysics, mechanoimmunology, mechanopathology, and mechanomedicine will promote the discovery of new strategies for immunomodulatory porous hydrogels in the coming years.

## Figures and Tables

**Table 1:** Summary of porous hydrogel types used for modulating immunity and tissue regeneration.

Strategies	Hydrogel	Pore Size (μm)	Gelation Mechanisms	Cell Type	Cellular Response	Refs.
Ice templating	Gelatin/PA	26–155	covalent interactions	Macrophages	Macrophages within smaller and softer pores exhibit proinflammatory phenotype, whereas anti-inflammatory phenotype is induced by larger and stiffer pores.	[33,38,40]
Pickering emulsions templating	GelMA	50–150	covalent interactions	BMSCs	Macroporous hydrogel speeds up stem cell migration to bone defects, promoting osteogenic differentiation and bone regeneration.	[48,51]
Microgel templating	Gelatin/GHS	10–100	covalent interactions	Osteoblast-like Saos-2 cells	A higher ratio of microgel-matrix would result in higher metabolic activity and a faster proliferation rate.	[54,57]
Phase separation	PEG and high viscous polysaccharides	0.5–50	noncovalent interactions	DRGs	The macroporous gels supported axonal growth in a rat sciatic nerve injury model.	[63]
Salt templating	HEMA copolymerized with EOEMA	185–485	covalent interactions	Osteoblast-like MG63 cells	The growth and survival of MG63 cells are mainly influenced by the higher elasticity of HEMA/EOEMA hydrogels and lack of positive charge, with pore size having a minimal impact.	[66]
Gas foaming	Gelatin	5–30	covalent interactions	L929	Macro-porous hydrogel can promote cell vitality and proliferation.	[69]
3D printing technique	GelMA	800–1200	covalent interactions	-	-	[74]
